# A descriptive analysis of a representative sample of pediatric randomized controlled trials published in 2007

**DOI:** 10.1186/1471-2431-10-96

**Published:** 2010-12-22

**Authors:** Michele P Hamm, Lisa Hartling, Andrea Milne, Lisa Tjosvold, Ben Vandermeer, Denise Thomson, Sarah Curtis, Terry P Klassen

**Affiliations:** 1 Alberta Research Centre for Health Evidence, Department of Pediatrics, University of Alberta. Edmonton, Canada; 2 Division of Pediatric Emergency Medicine, Department of Pediatrics, University of Alberta. Edmonton, Canada; 3 Women and Children's Health Research Institute, University of Alberta. Edmonton, Canada; 4 Manitoba Institute of Child Health, Winnipeg, Canada

## Abstract

**Background:**

Randomized controlled trials (RCTs) are the gold standard for trials assessing the effects of therapeutic interventions; therefore it is important to understand how they are conducted. Our objectives were to provide an overview of a representative sample of pediatric RCTs published in 2007 and assess the validity of their results.

**Methods:**

We searched Cochrane Central Register of Controlled Trials using a pediatric filter and randomly selected 300 RCTs published in 2007. We extracted data on trial characteristics; outcomes; methodological quality; reporting; and registration and protocol characteristics. Trial registration and protocol availability were determined for each study based on the publication, an Internet search and an author survey.

**Results:**

Most studies (83%) were efficacy trials, 40% evaluated drugs, and 30% were placebo-controlled. Primary outcomes were specified in 41%; 43% reported on adverse events. At least one statistically significant outcome was reported in 77% of trials; 63% favored the treatment group. Trial registration was declared in 12% of publications and 23% were found through an Internet search. Risk of bias (ROB) was high in 59% of trials, unclear in 33%, and low in 8%. Registered trials were more likely to have low ROB than non-registered trials (16% *vs*. 5%; *p *= 0.008). Effect sizes tended to be larger for trials at high *vs*. low ROB (0.28, 95% CI 0.21,0.35 *vs*. 0.16, 95% CI 0.07,0.25). Among survey respondents (50% response rate), the most common reason for trial registration was a publication requirement and for non-registration, a lack of familiarity with the process.

**Conclusions:**

More than half of this random sample of pediatric RCTs published in 2007 was at high ROB and three quarters of trials were not registered. There is an urgent need to improve the design, conduct, and reporting of child health research.

## Background

Randomized controlled trials (RCTs) are considered the gold standard for research on therapeutic interventions and provide the best evidence to inform and guide clinical decision-making. Currently the number of pediatric trials conducted and published lags behind that for adults [1,2]. In addition, little is known about the risk of bias, or validity, of pediatric RCTs.

Substantial evidence demonstrates that particular study design features increase the likelihood of systematic error, or bias, most often resulting in over-estimation of treatment effects. Risk of bias (ROB) reflects the degree to which the results of a trial should be believed [3]. Building on previous research around methodological quality of RCTs [4,5], the Cochrane Collaboration recently introduced a tool designed to appraise ROB, encompassing six domains related to the internal validity of a trial: sequence generation, allocation concealment, blinding, incomplete outcome data, selective outcome reporting, and "other" potential threats to validity [3].

Recent initiatives to address some of the biases associated with the design, conduct, and reporting of trials include the International Committee of Medical Journal Editors' (ICMJE) statement on trial registration [6] and reporting guidelines (http://www.equator-network.org) such as the CONSORT Statement (Consolidated Standards of Reporting Trials) [7]. Trial registration is integral in addressing the bias associated with selective outcome reporting by ensuring that investigators prospectively provide details on their trial, allowing for increased transparency and accountability [8]. The CONSORT Statement was developed to ensure adequate and transparent reporting upon completion of the trial and comprises a checklist of items that should be included in the publication of any RCT. Evidence suggests that these strategies have positively influenced the quality of published trials [9-11], but this has yet to be assessed in pediatrics.

Given these recent initiatives to improve reporting and assess ROB, we aimed to describe the state of pediatric evidence using a representative sample of child health RCTs published in 2007. Specific objectives were to examine: 1) methodological quality, including ROB, and its association with effect estimates; 2) the rate of trial registration and author reasons for registration and non-registration; and, 3) availability of trial protocols and their consistency with publications.

## Methods

### Sample Selection

Using a pediatric filter, the Cochrane Central Register of Controlled Trials (CENTRAL) was searched for trials published in 2007 [12]. CENTRAL is comprised of records of studies indexed in Medline and Embase, as well as hand-search results, grey literature, and the trials registers of Cochrane Review Groups [13]. As such, this provided a thorough search for pediatric controlled trials. Two thousand eight hundred thirty-two trials were randomly ordered using a computer-generated list, were screened consecutively for relevance, and the first 300 (approximately 10%) RCTs matching the criteria below were selected. Trials were included if they were published in English and included participants aged 0 to 18 years. If a trial studied both children and adults, it was included if the upper age limit was ≤21 years [13].

### Data Extraction

Data were extracted on: publication (e.g., type of journal, impact factor) and trial characteristics; outcomes and conclusions; methodological quality and reporting; and trial registration and protocol characteristics related to outcomes. Data extraction was completed by one reviewer with an independent second review on a randomly selected 10% sample. Discrepancies were resolved through consensus and were negligible. Trial registration and protocol availability were determined for each study based on publication details, an Internet search, and author follow-up.

### Assessment of Methodological Quality and Reporting

Given the range of quality assessment methods available, and the widespread use of many, methodological quality and reporting were assessed using multiple tools: the Jadad scale [4] and allocation concealment [5], as well as the Cochrane ROB tool [3] and the 2001 CONSORT Statement [14]. The Jadad scale is a five-point scale based on the description of randomization, double-blinding, and withdrawals or losses to follow-up; a score of 5 indicates highest quality. Allocation concealment was assessed as adequate, inadequate, or unclear. Nearly all trials in our sample were efficacy trials; therefore we focused on the original CONSORT Statement. The 2001 CONSORT checklist was the most recently published version at the time of data extraction, and assesses reporting with respect to 22 items. Each item was assessed as fully, partially, or not met.

The ROB tool was applied based on guidelines established by The Cochrane Collaboration [3], with some modifications specific to our investigation (see Additional file 1). These consist of decision rules that have been developed by our centre that have been used in conjunction with the Cochrane guidelines to increase consistency across reviewers. An overall assessment of ROB was made as high, low, or unclear based on the criteria from the Cochrane handbook: if any of the six domains were judged to be at high risk of bias, the overall risk was considered high; if any were judged to be at unclear risk of bias and none at high risk, the overall risk was unclear; and if all six domains were judged to be at low risk of bias, the overall risk was low. The tool was pilot tested by all members of the study team. Trials were assessed independently by two trained reviewers who arrived at consensus for each of the six items.

### Trial Registration and Protocol Availability

To determine whether or not trials were registered, details were first sought in the publication. If a declaration was not made, we searched through the International Clinical Trials Registry Platform (ICTRP) search portal maintained by the World Health Organization (WHO). If not found, the following registries were searched in order: ClinicalStudyResults.org, Memorial Sloan-Kettering Cancer Center, Current Controlled Trials Meta-Register, and CenterWatch. While there was some overlap in registries searched (i.e. ISRCTN.org is included in both the ICTRP portal and the Current Controlled Trials Meta-Register), each register contained unique databases. If a trial was not found in any of these registries, we conducted a Google search using the names of the first, last, and/or corresponding authors and key words. When available, data from the registry or from protocols found in our search were compared to the publication.

A 28-question survey regarding trial registration and protocol availability was sent to all corresponding authors with current email contact information (n = 290; see Additional file 2). The initial invitation and survey link was followed by two reminders containing the same information. Protocols were requested from authors. Ethical approval was obtained from the Health Research Ethics Board at the University of Alberta prior to survey implementation.

### Analysis

Data were analyzed descriptively, using means and standard deviations or medians and ranges for continuous variables and proportions for categorical variables. Effect sizes were computed for 236 trials with sufficient data based on the primary outcome for that trial. The effect size was a standardized mean difference when the primary outcome was continuous and a converted odds ratio when dichotomous [15]. Effect sizes were pooled using DerSimonian-Laird random effects for each of the three ROB categories (high, low, unclear). To compare ROB for certain covariates, a reference category was chosen within each variable classification and odds ratios comparing the number of high/unclear risk trials to low risk trials were computed with 95% confidence intervals.

## Results

### Description of Study Sample

Publication and trial characteristics of our sample of 300 trials are shown in Table 1. The majority of trials used parallel designs (89.7%), were efficacy trials (82.7%), and were published in specialty journals (78.6%). Evaluation of pharmacological interventions was most common (40.3%) and 30% of trials were placebo-controlled. While all major geographic areas were represented, the majority of authors were from Europe (40.3%) and North America (29.0%). Each study was categorized using the review groups of The Cochrane Collaboration: neonatal (9.3%), oral health (7.7%), and developmental, psychosocial, and learning problems (6.7%) were most represented.

**Table 1:** Publication and trial characteristics (N = 300)

Study Characteristic	N (%)
Continent of corresponding author	
Africa	11 (3.7)
Asia	58 (19.3)
Australia	16 (5.3)
Europe (excluding UK)	91 (30.3)
North America	87 (29.0)
South America	7 (2.3)
United Kingdom	30 (10.0)

Type of journal	
General medical journal	19 (6.3)
Specialty medical journal	166 (55.3)
General pediatric journal	45 (15.0)
Specialty pediatric journal	70 (23.3)

Study design	
RCT parallel	269 (89.7)
RCT crossover	19 (6.3)
RCT factorial	5 (1.7)
Other	7 (2.3)

Study type	
Efficacy/Superiority	248 (82.7)
Equivalence	9 (3.0)
Non-inferiority	13 (4.3)
Not declared	2 (0.7)
None of the above	25 (8.3)
Unclear	3 (1.0)

Nature of intervention	
Drug	121 (40.3)
Vaccine	16 (5.3)
Natural health product	26 (8.7)
Device	44 (14.7)
Other	93 (31.0)

Placebo-controlled	90 (30.0)

Number of centres	
Multicentre	105 (35.0)
Single Centre	179 (59.7)
Unclear	16 (5.3)

Sample size	
Mean (SD)	785.2 (5837.3)
Median (range), IQR	83 (6 - 71,799), 10 - 7079

Data Monitoring Committee established	14 (4.7)

Any adverse events reported	129 (43.0)

Funding source	
Declared	194 (64.7)
Industry Sponsored	67/194 (34.5)

Primary outcome explicitly reported	123 (41.0)

At least one statistically significant outcome	230 (76.7)

Intervention favored	
Treatment	189 (63.0)
Control	19 (6.3)
Neither	92 (30.7)

Common primary diagnostic categories	
Acute Respiratory Infections	17 (5.7)
Airways	14 (4.7)
Anaesthesia	18 (6.0)
Developmental, Psychosocial, and Learning Problems	20 (6.7)
Ear, Nose, and Throat Disorders	10 (3.3)
Infectious Disease	19 (6.3)
Metabolic and Endocrine Disorders	15 (5.0)
Neonatal	28 (9.3)
Oral Health	23 (7.7)
Public Health	16 (5.3)

### Methodological Quality

The median Jadad score was 2 (IQR 2-3). Allocation concealment was adequate in 21.7% of trials, while 75.7% were unclear (Table 2). Only three trials (1.0%) sufficiently addressed all 22 items of the CONSORT Statement (IQR 13-17) with another eight (2.7%) at least partially meeting all requirements (IQR 15-19). The remaining 289 trials (96.3%) failed to report at least one, and up to 14 recommended items. Overall, the median number of items that were adequately addressed was 15, and five for those partially addressed. Descriptions of the "method used to implement the randomization sequence" (item 9) and "who generated the allocation sequence and enrolled and assigned participants" (item 10) were the most under-reported, with 214 (71.3%) and 229 (76.3%) trials not meeting these criteria respectively.

**Table 2:** Assessments of methodological quality (N = 300)

Methodological Quality Indicator	N (%)
Jadad	
Mean (SD)	2.6 (1.2)
Median (range)	2 (0 - 5)

Allocation Concealment	
Adequate	65 (21.7)
Unclear	227 (75.7)
Inadequate	8 (2.7)

Risk of Bias	
Low	23 (7.7)
Unclear	99 (33.0)
High	178 (59.3)

CONSORT Statement	
Items fully addressed (median, range)	15 (4-22)
Items partially addressed (median, range)	5 (0-14)
Items not addressed (median, range)	2 (0-7)

Trial registered	
Declared in publication	37 (12.3)
Registration found online	69 (23.0)

Study protocol available from corresponding author	2/290 (0.7)

Overall ROB was low for 23 trials (7.7%), unclear for 99 (33.0%), and high for 178 (59.3%) (Table 2). Much of the uncertainty in rating studies was due to unclear reporting. Selective outcome reporting was rated as low ROB in nearly all trials. "Other" sources of bias included inappropriate influence of the study sponsor (e.g. industry funding without separation from the conduct of the trial), imbalance in baseline characteristics, and design-specific issues (e.g., factors related to cluster RCTs or cross-over trials), and was the domain that was least often addressed satisfactorily (Table 3). Trials at low ROB had higher mean Jadad scores and were more likely to report adequate means of allocation concealment than those at high ROB (Table 4).

**Table 3:** Risk of bias assessments by domain (N = 300)

Domain	Risk of bias assessments - N (%)		
	**High**	**Unclear**	**Low**

Sequence generation	8 (2.7%)	143 (47.7%)	149 (49.7%)

Allocation concealment	8 (2.7%)	217 (72.3%)	75 (25.0%)

Blinding	41 (13.7%)	108 (36.0%)	151 (50.3%)

Incomplete data	60 (20.0%)	53 (17.7%)	187 (62.3%)

Selective reporting	48 (16.0%)	6 (2.0%)	246 (82.0%)

"Other" sources of bias	85 (28.3%)	109 (36.3%)	106 (35.3%)

**Table 4:** Trial characteristics and quality assessment stratified by trial registration and overall risk of bias (N = 300)

Trial Characteristics	N	Trial Registered		Risk of Bias		
	**300**	**Yes (N = 69;23%)**	**No (N = 231;77%)**	**Low (N = 23;8%)**	**Unclear (N = 99;33%)**	**High (N = 178;59%)**

Impact factor (median, range)	294	4.017 (0.581-52.589)	1.883 (0.080-15.484)	2.948 (0.475-10.169)	1.850 (0.329-52.589)	2.342 (0.080-28.638)

Continent of corresponding author						
Africa	11	4 (5.8)	7 (3.0)	1 (4.4)	4 (4.0)	6 (3.4)
Asia	58	6 (8.7)	52 (22.5)	6 (26.1)	27 (27.3)	25 (14.0)
Australia	16	1 (1.5)	15 (6.5)	2 (8.7)	7 (7.1)	7 (3.9)
Europe (excluding UK)	91	17 (24.6)	74 (32.0)	6 (26.1)	27 (27.3)	58 (32.6)
North America	87	30 (43.5)	57 (24.7)	5 (21.7)	19 (19.2)	63 (35.4)
South America	7	2 (2.9)	5 (2.2)	-	2 (2.0)	5 (2.8)
United Kingdom	30	9 (13.0)	21 (9.1)	3 (13.0)	13 (13.1)	14 (7.9)

Funding source specified	194	62 (89.9)	132 (57.1)	23 (100.0)	49 (49.5)	122 (68.5)

Industry supported	67	24/62 (38.7)	43/132 (32.6)	7/23 (30.4)	8/49 (16.3)	52/122 (42.6)

Primary outcome explicitly stated	123	41 (59.4)	82 (35.5)	14 (60.9)	34 (34.3)	75 (42.1)

Statistically significant outcome	230	47 (68.1)	183 (79.2)	15 (65.2)	83 (83.8)	132 (74.2)

Data Monitoring Committee	14	9 (13.0)	5 (2.2)	1 (4.4)	5 (5.1)	8 (4.5)

Jadad score (mean; median, range)	300	2.99 (3; 0-5)	2.44 (2; 0-5)	3.96 (4; 3-5)	2.24 (2; 1-5)	2.56 (2; 0-5)

Allocation Concealment						
Adequate	65	24 (34.8)	41 (17.8)	20 (87.0)	15 (15.2)	30 (16.9)
Unclear	227	45 (65.2)	182 (78.8)	3 (13.0)	84 (84.9)	140 (78.7)
Inadequate	8	-	8 (3.5)	-	-	8 (4.5)

Trial registered	69	NA	NA	11 (47.8)	10 (10.1)	48 (27.0)

Risk of Bias						
Low	23	11 (15.9)	12 (5.2)	NA	NA	NA
Unclear	99	10 (14.5)	89 (38.5)			
High	178	48 (69.6)	130 (56.3)			

**Figure 1:**
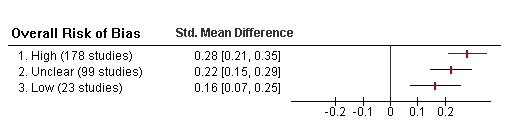
**Effect size estimates according to overall risk of bias**.

Effect sizes tended to increase from studies at low (0.16, 95% CI 0.07,0.25) to high ROB (0.28, 95% CI 0.21,0.35; *p *= 0.051; Figure 1).

Each of the ROB domains and the overall ratings were examined in the context of the following variables: trial registration, industry funding, multi-centre status, number of treatment arms, intervention type, primary outcome category, and type of journal (see Additional file 3). Of these variables, trial registration had the most influence on ROB. Compared to trials that were not registered, those trials that were had a lower overall ROB, as well as a lower ROB for each of the domains except selective outcome reporting. Odds ratios for high ROB ranged from 0.29 (95% CI 0.12,0.69) for overall ROB to 0.47 (95% CI 0.27,0.81) for "other" sources of bias. Trials that were sponsored by industry were more likely to have adequate blinding than non-industry funded trials (OR 0.41 (95% CI 0.22,0.76)), but were also more likely to be associated with "other" sources of bias (OR 4.72 (95% CI 2.46,9.07)). ROB for selective outcome reporting increased with number of arms in the trial (*p *= 0.007), but was unchanged for the other domains. When compared to pharmacological interventions, trials investigating devices had a higher ROB associated with blinding (OR 3.37 (95% CI 1.62,7.02)) and incomplete data (OR 2.56 (95% CI 1.26,5.21)). High ROB due to blinding was also found in studies with outcomes related to techniques/training (e.g., longevity of dental restorations) when compared to physiological outcomes (OR 5.28 (95% CI 1.09,25.61)). Multi-centre status and type of journal had no impact on ROB.

Trial registration was declared in the publication of 37 trials (12.3%) and 69 records of registration (23.0%) were found online. Registered trials were more likely to be published in journals with a higher impact factor (median 4.017 *vs*. 1.883; *p *< 0.0001). Approximately one third of trials were registered in studies with corresponding authors from Africa (36.4%), North and South America (34.5% and 28.6%, respectively), and the UK (30.0%), but proportions were lower for the rest of Europe (18.7%), Asia (10.3%), and Australia (6.3%). Registered trials more often specified their funding source (89.9% vs. 57.1%; *p *< 0.0001), and less often reported statistically significant findings, although this comparison was not statistically significant (68.1% *vs*. 79.2%; *p *= 0.07). Measures of methodological quality were superior in registered trials (Table 4).

### Author Follow-Up Survey

145 authors (50.0%) responded to the survey, therefore the ability to generalize findings is limited. Of respondents, 61 (42.4%) reported registration with a public trial registry, potentially corresponding closely to the 69 found in our search. The majority of these were registered with ClinicalTrials.gov (67.5%) or Current Controlled Trials (17.5%). 51.2% were registered prior to and 37.2% after patient recruitment. The most common reason for registering a trial was a journal requirement for publication (72.7%), followed by a belief in full public disclosure (68.2%). For non-registration, the most common reasons were lack of familiarity with the process (59.1%) and trial initiation prior to registration endorsement by the ICMJE (51.5%) (Table 5).

**Table 5:** Author responses to follow-up survey (N = 145)

Survey Question	N (%)
*Was your trial registered with a public trial registry?*	
Yes	61 (42.4)
No	83 (57.6)
No response	1

*What were your reasons for registering your trial (select all that apply)?*	
I believe that trials should be registered as a means of full public disclosure	30 (68.2)
I endorse the statement regarding public trial registration made by the ICJME	23 (52.3)
Trial registration is necessary for publication in some peer-reviewed journals	32 (72.7)
Trial registration was required by the funding agency	5 (11.4)
Trial registration was required by the Research Ethics Board	9 (20.5)
Trial registration is institutional policy	2 (4.5)
Other	3 (6.8)
No response	101

*What were your reasons for not registering your trial (select all that apply)?*	
Lack of time	3 (4.5)
Lack of resources	5 (7.6)
I was not familiar with the process for trial registration	39 (59.1)
Cost associated with registration	4 (6.1)
I don't see a benefit to trial registration	1 (1.5)
Trial was initiated prior to registration endorsement by the ICMJE	34 (51.5)
No formal requirement	4 (6.1)
Other	7 (10.6)
No response	79

Nearly all respondents (92.2%) had prepared a study protocol prior to trial initiation; 2.0% reported a minor difference between the protocol and study conduct. 9.7% of authors reported that some outcomes measured in the trial were not reported in the publication. Space limitations were the most common concern (journal imposed space limitation 41.7%; authors' concern about space 25.0%), followed by non-statistically significant findings (41.7%). While 56.4% of respondents indicated that they were willing to share their protocol, only two were received. In both cases, the details in the publication were consistent with the protocol.

## Discussion

Our sample of recently-published pediatric trials demonstrates that there is considerable room for improvement in their design, conduct, and reporting. Methodological quality was modest, with the vast majority of trials at high or unclear ROB. Further, the trials did not adhere to widely accepted reporting standards or requirements for trial registration.

Our sample was intended to be representative of all RCTs published in 2007; therefore we placed no restrictions on journal, clinical area, or type of intervention. Trials in our sample were largely published in specialty journals, and examined a variety of interventions among a diverse range of conditions.

Methodological quality was assessed using three well-recognized tools and the results overall were not favorable, suggesting methodological weaknesses and high risk of bias. Incomplete reporting was prevalent; while statements declaring implementation of certain design features (e.g., randomization and "double-blinding") were common, detailed methods were often not specified. Further, allocation concealment was rarely addressed at all. Despite the differing emphasis of the tools used (i.e., conduct for ROB and quality of reporting for Jadad and CONSORT), the results were consistent in that overall, the trials did not meet the criteria of any of the methods of assessment. However, there is evidence to suggest that the Jadad scale and ROB measure different constructs and that the assessment of ROB may be more appropriate [16].

Selective outcome reporting is of great concern. It is one of the driving forces for the promotion of trial registration and has important implications for safety [17-20]. To assess this domain, we compared the outcomes specified in the protocol or in the trial register to those reported in the publication; however the lack of registered trials and the extremely low response to requests for protocols made this difficult. As a result, our findings likely underestimate the risk associated with this particular issue, as we were unable to assess potential biases introduced through discrepancies between the original trial design and actual conduct.

Evidence suggests that industry-funded trials are more likely to report favorable results [21-23], therefore we included a criterion within the "other" sources of bias domain that related to inappropriate influence of the funding body. Provided that the source of funding was declared and a statement was made outlining the role of the sponsor, we considered the trial to be low ROB for that measure; however this information was often missing. While funding source was not the only consideration in assessing "other" sources of bias, it was relevant to every trial, and was therefore important in the determination of our overall results showing high or unclear ROB for this domain among two thirds of trials.

We found a noteworthy trend toward increasing effect estimates with increasing ROB which is consistent with previous research [16]. Trials at high ROB had a larger mean effect size than trials at low ROB, indicating the potential for a high proportion of trials to be reporting exaggerated results. These results are exploratory and should be interpreted with caution given the heterogeneity in outcomes compared and the small number of studies. Further work and methods that better account for confounding due to intervention and diagnostic condition are required before firm conclusions can be made.

Despite wide support [24], uptake by journals of the CONSORT Statement has been variable. In a survey of 165 high impact journals in 2007, 38% mentioned the CONSORT Statement in the instructions to authors and 14% required (rather than recommended) it to be completed for a trial to be accepted [25]. This variability is echoed in our sample, as very few trials met all of the requirements of the checklist. Of the 11 trials that at least partially met all requirements, nine journals were represented. Of these, two journals stated in their instructions to authors that a completed CONSORT checklist was required, three recommended following the CONSORT guidelines, and the remainder did not mention the CONSORT Statement. Our observation that journal endorsement of the CONSORT Statement has little bearing on whether all of the recommended elements are reported highlights the practical issue of how to ensure adherence to the guidelines, and ultimately their impact on reporting.

Very few trials in our sample were registered in a public registry, and only about half of those that were registered declared this in the publication. Prospective trial registration has been heavily endorsed, and the volume of trials registered appears to be increasing [26-28]. However, trial registration is far from universal, and is perhaps more problematic in pediatric trials. Pandolfini and Bonati [29] found that the proportion of pediatric trials among all registered trials in online registers was disproportionate to those in the published literature. Pediatric trials are more likely to be published in specialty journals which may be less likely to require trial registration than general medical journals. Another concern is that the requirement for trial registration may not be enforced. Our author survey suggests that one of the major barriers to trial registration among respondents is a lack of familiarity with the process; therefore, efforts are required to raise awareness. These efforts should target researchers at the study design stage, rather than at the point of publication. However, reluctance on the part of academic researchers to publicly disclose the information required by trial registers may pose a challenge [30], an issue that was reinforced in this study by the apparent futility of contacting authors for access to protocol data. Potential future directions in this area may include the requirement of publicly available protocols at the time of trial registration or with funding applications.

Based on our findings, there is clearly room for improvement in pediatric trials. This is the mission of StaR Child Health (Standards for Research in Child Health), an international group that was recently formed involving varied stakeholders to develop and promote guidance to ensure the validity and relevance of pediatric trials [31]. With the involvement of trialists, clinicians, regulators, editors, and representatives of the pharmaceutical industry, this initiative is invested in meeting the needs of the research and clinical communities [32]. Through the development of standards for research in priority areas for pediatric research (e.g. appropriate outcome selection, data and safety monitoring committees, sample size, ROB), StaR Child Health aims to be an important contributor to a methodologically strong evidence base for pediatric care [33].

### Limitations

We included approximately 10% of pediatric RCTs published in 2007, potentially limiting representativeness. Only trials published in English were included, possibly contributing to the high proportion of studies from North America and the UK. While we extracted the country of the corresponding author, this is not a perfect proxy for the population studied and in some cases, an author from a high income country reported on a trial conducted in a low or middle income area.

The true ROB was often difficult to interpret in our sample due to poor reporting. Additionally, the issue of selective outcome reporting posed a challenge as protocols or trial registers were unavailable for the majority of studies. In most cases, the publication was judged according to its internal consistency. Hence, the high proportion of trials that were given a rating of low ROB for this domain likely underestimates the true ROB.

The pooled analysis presented to examine trends in effect sizes and ROB is preliminary work. Given the heterogeneity in diseases, interventions, and outcomes included in the sample, we used standardized measures of effect size to be able to investigate general patterns across studies, but these results are exploratory.

The response to our author survey was likely subject to response bias. The item responses indicate that authors who had registered their trials were more likely to participate in the survey, potentially limiting applicability. Assuming that respondents were more aware of issues related to trial registration and methodological initiatives in general, the answers provided (for example reasons for non-registration) may not encompass some of the deeper issues faced by other researchers and may have implications for attempts to overcome these barriers in the future.

## Conclusions

This study shows that the majority of pediatric trials published in 2007 were at high risk of bias, corresponding with a trend toward increased effect sizes. In spite of a movement towards improving methodological quality and requirements for trial registration, the majority of trials have not met these recommendations. These results should be of great concern for child health providers, researchers, methodologists, and funders, and should motivate all to work towards improving the design, conduct, and reporting of child health research.

## Competing interests

The authors declare that they have no competing interests.

## Authors' contributions

MPH contributed to study design, coordinated the study, extracted data, performed quality and risk of bias assessments, analyzed data, and drafted the manuscript. LH contributed to study design, provided oversight for the study and analysis, contributed to interpretation of data, helped draft and edit the manuscript, and is guarantor. AM extracted data, conducted quality and risk of bias assessments, and critically reviewed the manuscript. LT performed the literature search. BV performed statistical analysis and interpreted the data. DT and SC contributed to study design and critically reviewed the manuscript. TPK contributed to study design, provided oversight for the study, and critically reviewed the manuscript. All authors gave final approval of the version to be published.

## Pre-publication history

The pre-publication history for this paper can be accessed here:

http://www.biomedcentral.com/1471-2431/10/96/prepub

## Supplementary Material

Additional file 1 **Guidelines and Decision Rules for Risk of Bias Assessments**. List of decision rules developed by our research group to be used with the Cochrane Handbook in assessing risk of bias. Click here for file

Additional file 2 **Author Follow-Up Survey**. Included questions in the survey sent to 290 corresponding authors. Click here for file

Additional file 3 **Odds Ratios for Risk of Bias by Selected Variables**. Odds ratios for high risk of bias by selected variables, stratified by the six domains of the Cochrane Collaboration's Risk of Bias tool. Click here for file
