# Supplementary material for: A descriptive analysis of a representative sample of pediatric randomized controlled trials published in 2007
Source: BMC Pediatr. 2010 Dec 22;10:96. doi: 10.1186/1471-2431-10-96 (PMC3018376; doi:10.1186/1471-2431-10-96)
Supplement: Additional file 1 — Guidelines and Decision Rules for Risk of Bias Assessments. List of decision rules developed by our research group to be used with the Cochrane Handbook in assessing risk of bias. [file 1471-2431-10-96-S1.DOC]

**Additional file 1: Guidelines and Decision Rules for Risk of Bias Assessments**

*Use these decision rules in addition to the guidelines outlined in the Cochrane criteria.

**Sequence generation:**

- If blocked randomization, permutation, or stratification is specified, assume the randomization sequence was computer-generated and answer YES.
- If the description only includes ‘random’, ‘randomly generated’, ‘randomized’, etc, do not assume additional details and answer UNCLEAR.

**Allocation concealment:**

- If the randomization is conducted by central telephone, pharmacy, etc, assume this is adequate and answer YES.

**Blinding:**

- Determine whether the blinding is likely to be broken, and whether the outcomes in unblinded studies are likely to be influenced by the lack of blinding.
- If a study is described as “double-dummy”, assume that this is appropriate and answer YES. If it is described as “double-blind” without further details, answer UNCLEAR.

**Incomplete outcome data:**

- Look for intention-to-treat analysis. If this was done appropriately, answer YES.
- If all participants were accounted for (i.e. no drop-outs or censored analysis conducted), answer YES.
- If the numbers and reasons for withdrawal/drop-outs were described and comparable across groups (and ≤ approximately 10%), answer YES.
- If there is greater than 10% drop-out, consider UNCLEAR or NO.

**Selective outcome reporting:**

- If the study protocol is available, compare the outcomes reported in the publication to those specified in the protocol. Answer YES if the outcomes in the two documents match.
- If the study protocol is not available, compare the outcomes reported in the Methods and Results sections. Answer YES if these match.

**“Other” sources of bias:**

- Assess for baseline imbalances that could have biased the results (or were not accounted for).
- Assess for early stopping for benefit.
- Assess for appropriateness of cross-over design (e.g. inadequate wash-out period).
- Assess for inappropriate influence of funders that could have biased the results.
  - If sponsor is acknowledged and there is a clear statement regarding no involvement of sponsor in trial conduct or data management/analysis, answer YES.
  - If sponsor is acknowledged and no one from the sponsoring agency was an author, answer YES.
  - If sponsor is acknowledged and someone from the sponsoring agency was an author, answer NO.
  - If a drug/intervention is provided by industry, but the trial has no other inappropriate influence of funding, answer YES.
  - If there is no mention of funding source, answer UNCLEAR.
- Note any “other” sources of bias.
