# Supplementary material for: A descriptive analysis of a representative sample of pediatric randomized controlled trials published in 2007
Source: BMC Pediatr. 2010 Dec 22;10:96. doi: 10.1186/1471-2431-10-96 (PMC3018376; doi:10.1186/1471-2431-10-96)
Supplement: Additional file 2 — Author Follow-Up Survey. Included questions in the survey sent to 290 corresponding authors. [file 1471-2431-10-96-S2.DOC]

**Additional file 2: Author Follow-Up Survey**

*Skip logic was included to automatically bypass irrelevant questions (e.g. a response of “no” for #1 would skip #2-4).

*1. Was your study registered with a public trial registry?*

- Yes

- No

**2. Trial Registration**

*2. Where is your trial registered (select all that apply)?*

- Australian New Zealand Clinical Trials Registry (ANZCTR)

- Chinese Clinical Trial Register

- ClinicalTrials.gov

- Clinical Trials Registry - India

- Current Controlled Trials (ISRCTN Register)

- German Clinical Trials Register

- Iranian Registry of Clinical Trials

- Sri Lanka Clinical Trials Registry

- The Netherlands National Trial Register

- Other (please specify)

*3. When was your trial registered?*

- Before patient recruitment

- During patient recruitment

- After patient recruitment

*4. What were your reasons for registering your trial (select all that apply)?*

- I believe that trials should be registered as a means of full public disclosure

- I endorse the statement regarding public trial registration made by the International Committee of Medical Journal Editors

- Trial registration is necessary for publication in some peer-reviewed journals

- Trial registration was required by the funding agency

- Trial registration was required by the Research Ethics Board

- Other (please specify)

**3. Trial Registration**

*5. What were your reasons for not registering your trial (select all that apply)?*

- Lack of time

- Lack of resources

- I was not familiar with the process for trial registration

- Cost associated with registration

- I don't see a benefit to trial registration

- Trial was initiated prior to registration endorsement by the International Committee of Medical Journal Editors

- Other (please specify)

**4. Study Protocol**

*6. Did you prepare a formal, written, study protocol prior to implementing your trial?*

- Yes

- No

**5. Study Protocol**

*7. Is your protocol publicly available (e.g. on a publicly available website, published in a journal)?*

- Yes

- No

- If yes, where is it located?

*8. If your protocol is not publicly available, would you be willing to share it with us? All information will be kept confidential, will only be used for the purposes of this study, and will be reported on in aggregate form.*

- No

- Yes (please send to study coordinator as per the contact information in the cover letter)

- Yes, I would be willing to share it but I cannot locate it

- Other (please specify)

*9. Did the study conduct differ from that specified in the protocol?*

- Yes

- No

**6. Study Protocol**

*10. How would you rate the difference in study conduct from that indicated in the protocol?*

- Major difference

- Minor difference

*11. How did the study conduct differ from that specified in the protocol (select all that apply)?*

- Recruitment of participants

- Sample size

- Intervention in the treatment arm

- Intervention in the control arm

- Outcome measurement

- Monitoring of safety and efficacy data

- Duration of study

- Length of follow-up

- Analysis

- Other (please specify)

**7. Study Methodology**

*12. What was the primary outcome measure(s) in your study?*

*13. When was the primary outcome specified?*

- In the protocol before the trial began

- In the protocol during the trial

- During data analysis

- Following data analysis

- Other (please specify)

**8. Study Methodology**

*14. Please list any outcomes that were measured in the trial, but were not reported in the publication. Please exclude baseline characteristics and data collected for administrative purposes.*

*15. For each outcome listed in Question 14, indicate whether or not the result was statistically significant (p≤0.05).*

*16. For each outcome listed in Question 14, indicate whether it was a primary, secondary, or unspecified outcome in the protocol.*

*17. For each outcome listed in Question 14, indicate whether it was of little, moderate, or high clinical importance.*

*18. What were your reasons for not reporting the above outcomes (select all that apply)?*

- Journal imposed space limit

- Authors' concern about space

- Not statistically significant

- Not clinically important

- Not intended for inter-group comparisons

- Other (please specify)

*19. Were outcomes measured at any timepoints that were not reported on?*

- Yes

- No

- If yes, please specify

**9. Study Methodology**

*20. Which study type is consistent with your trial?*

- Efficacy/superiority

- Equivalence

- Non-inferiority

- None of the above (please specify)

*21. Would you consider the results of your trial to be:*

- Positive

- Negative

- Null

- Unclear

*22. Did you conduct sample size calculations before the study began?*

- Yes

- No

*23. If yes, which outcome(s) was the sample size calculation based on?*

*24. Prior to the publication of your trial, was your manuscript ever refused by a journal because the trial hadn't been registered?*

- Yes

- No

- Not Applicable

**10. Demographic Information**

*25. Which most closely describes your academic rank when the trial was initiated?*

- Research Assistant

- Research Associate

- Lecturer

- Assistant Professor

- Associate Professor

- Full Professor

- Other (please specify)

*26. How many trials (RCTs) had you previously completed at the time that this trial was initiated?*

*27. What is your gender?*

- Female

- Male

*28. What was your age at the time that this trial was initiated?*

- Less than 30 years

- 30 - 39 years

- 40 - 49 years

- 50 - 59 years

- 60 years or more
