# Supplementary material for: A descriptive analysis of a representative sample of pediatric randomized controlled trials published in 2007
Source: BMC Pediatr. 2010 Dec 22;10:96. doi: 10.1186/1471-2431-10-96 (PMC3018376; doi:10.1186/1471-2431-10-96)
Supplement: Additional file 3 — Odds Ratios for Risk of Bias by Selected Variables. Odds ratios for high risk of bias by selected variables, stratified by the six domains of the Cochrane Collaboration's Risk of Bias tool. [file 1471-2431-10-96-S3.DOC]

**Additional file 3: Odds Ratios for Risk of Bias by Selected Variables**

**Table A1. High Risk of Bias by Sequence Generation (N=300)**

| **Variable** | **Sequence Generation** | |
| --- | --- | --- |
|  | **OR (95%CI)** | ***p-*value** |
| Trial registration | 0·44 (0·25,0·76) | <0·01 |
| Industry funding | 1·24 (0·68,2·25) | 0·48 |
| Multi-centre trial | 1·24 (0·77,2·02) | 0·38 |
| Type of journal  Specialty medical  General medical  Specialty pediatric  General pediatric | Reference  1·36 (0·52,3·55)  1·11 (0·63,1·94)  0·79 (0·41,1·53) | 0·53  0·72  0·49 |
| Nature of intervention  Drug  Device  Natural health product  Vaccine  Other | Reference  1·03 (0·52,2·07)  0·78 (0·33,1·85)  1·60 (0·56,4·56)  2·06 (1·18,3·57) | 0·92  0·57  0·38  0·01 |
| Outcome category  Physiological  Behavioural  Biomarker  Pain  Psychological  Quality of life  Techniques/Training  Other | Reference  1·46 (0·71,3·01)  1·53 (0·75,3·15)  0·96 (0·38,2·43)  1·56 (0·52,4·69)  8·17 (0·42,160·72)  0·78 (0·21,2·86)  2·63 (0·78,8·88) | 0·30  0·24  0·93  0·43  0·17  0·71  0·12 |

**Table A2. High Risk of Bias by Allocation Concealment (N=300)**

| **Variable** | **Allocation Concealment** | |
| --- | --- | --- |
|  | **OR (95%CI)** | ***p-*value** |
| Trial registration | 0.31 (0·18,0·56) | <0·01 |
| Industry funding | 1·17 (0·61,2·25) | 0·63 |
| Multi-centre trial | 1·34 (0·76,2·37) | 0·31 |
| Type of journal  Specialty medical  General medical  Specialty pediatric  General pediatric | Reference  0·58 (0·21,1·65)  0·84 (0·43,1·63)  0·49 (0·24,1·00) | 0·31  0·60  0·05 |
| Nature of intervention  Drug  Device  Natural health product  Vaccine  Other | Reference  1·56 (0·70,3·47)  0·86 (0·35,2·12)  1·37 (0·42,4·54)  2·58 (1·30,5·13) | 0·28  0·75  0·60  <0·01 |
| Outcome category  Physiological  Behavioural  Biomarker  Pain  Psychological  Quality of life  Techniques/Training  Other | Reference  1·84 (0·72,4·73)  1·34 (0·57,3·14)  0·55 (0·21,1·44)  1·35 (0·36,5·07)  0·74 (0·07,8·33)  1·48 (0·30,7·21)  2·03 (0·43,9·51) | 0·20  0·51  0·23  0·65  0·81  0·63  0·37 |

**Table A3. High Risk of Bias by Blinding (N=300)**

| **Variable** | **Blinding** | |
| --- | --- | --- |
|  | **OR (95%CI)** | ***p-*value** |
| Trial registration | 0·45 (0·26,0·79) | 0·01 |
| Industry funding | 0·41 (0·22,0·76) | <0·01 |
| Multi-centre trial | 1·16 (0·72,1·88) | 0·54 |
| Type of journal  Specialty medical  General medical  Specialty pediatric  General pediatric | Reference  1·27 (0·49,3·29)  1·62 (0·92,2·84)  0·91 (0·47,1·77) | 0·62  0·10  0·79 |
| Nature of intervention  Drug  Device  Natural health product  Vaccine  Other | Reference  3·37 (1·62,7·02)  0·70 (0·28,1·74)  0·36 (0·10,1·34)  3·00 (1·71,5·27) | <0·01  0·70  0·13  <0·01 |
| Outcome category  Physiological  Behavioural  Biomarker  Pain  Psychological  Quality of life  Techniques/Training  Other | Reference  1·85 (0·89,3·83)  1·55 (0·76,3·17)  1·98 (0·77,5·09)  0·99 (0·33,2·98)  0·66 (0·06,7·42)  5·28 (1·09,25·61)  2·97 (0·88,10·03) | 0·10  0·23  0·16  0·99  0·74  0·04  0·08 |

**Table A4. High Risk of Bias by Incomplete Data (N=300)**

| **Variable** | **Incomplete Data** | |
| --- | --- | --- |
|  | **OR (95%CI)** | ***p-*value** |
| Trial registration | 0·46 (0·25,0·84) | 0·01 |
| Industry funding | 0·82 (0·43,1·56) | 0·55 |
| Multi-centre trial | 1·12 (0·68,1·85) | 0·65 |
| Type of journal  Specialty medical  General medical  Specialty pediatric  General pediatric | Reference  0·95 (0·34,2·63)  1·63 (0·92,2·90)  0·91 (0·47,1·77) | 0·92  0·09  0·09 |
| Nature of intervention  Drug  Device  Natural health product  Vaccine  Other | Reference  2·56 (1·26,5·21)  2·99 (1·25,7·10)  0·59 (0·16,2·20)  1·93 (1·09,3·42) | 0·01  0·01  0·43  0·02 |
| Outcome category  Physiological  Behavioural  Biomarker  Pain  Psychological  Quality of life  Techniques/Training  Other | Reference  2·10 (1·01,4·35)  1·02 (0·48,2·15)  1·25 (0·48,3·24)  0·51 (0·14,1·91)  3·76 (0·33,42·33)  1·88 (0·52,6·76)  1·17 (0·37,3·75) | 0·05  0·96  0·64  0·32  0·28  0·33  0·79 |

**Table A5. High Risk of Bias by Selective Outcome Reporting (N=300)**

| **Variable** | **Selective Outcome Reporting** | |
| --- | --- | --- |
|  | **OR (95%CI)** | ***p-*value** |
| Trial registration | 3·23 (1·72,6·04) | <0·01 |
| Industry funding | 1·36 (0·64,2·91) | 0·43 |
| Multi-centre trial | 1·24 (0·67,2·30) | 0·49 |
| Type of journal  Specialty medical  General medical  Specialty pediatric  General pediatric | Reference  1·83 (0·61,5·49)  0·95 (0·44,2·05)  1·46 (0·65,3·30) | 0·28  0·90  0·36 |
| Nature of intervention  Drug  Device  Natural health product  Vaccine  Other | Reference  0·37 (0·12,1·11)  1·10 (0·40,3·01)  0·24 (0·03,1·93)  0·82 (0·41,1·62) | 0·08  0·86  0·18  0·56 |
| Outcome category  Physiological  Behavioural  Biomarker  Pain  Psychological  Quality of life  Techniques/Training  Other | Reference  1·10 (0·44,2·75)  0·88 (0·34,2·31)  1·14 (0·36,3·66)  0·76 (0·16,3·58)  9·13 (0·80,104·03)  0·21 (0·01,3·76)  1·37 (0·36,5·28) | 0·83  0·80  0·82  0·73  0·07  0·29  0·65 |

**Table A6. High Risk of Bias by “Other” Sources of Bias (N=300)**

| **Variable** | **“Other” Sources of Bias** | |
| --- | --- | --- |
|  | **OR (95%CI)** | ***p-*value** |
| Trial registration | 0·47 (0·27,0·81) | 0·01 |
| Industry funding | 4·72 (2·46,9·07) | <0·01 |
| Multi-centre trial | 1·28 (0·77,2·13) | 0·34 |
| Type of journal  Specialty medical  General medical  Specialty pediatric  General pediatric | Reference  0·60 (0·23,1·57)  1·45 (0·79,2·69)  0·74 (0·38,1·45) | 0·30  0·23  0·38 |
| Nature of intervention  Drug  Device  Natural health product  Vaccine  Other | Reference  0·82 (0·39,1·71)  0·68 (0·28,1·64)  2·96 (0·64,13·72)  0·49 (0·28,0·87) | 0·59  0·39  0·16  0·01 |
| Outcome category  Physiological  Behavioural  Biomarker  Pain  Psychological  Quality of life  Techniques/Training  Other | Reference  0·90 (0·43,1·90)  1·36 (0·63,2·94)  1·73 (0·60,4·98)  0·77 (0·25,2·32)  1·15 (0·10,12·96)  1·34 (0·33,5·38)  0·92 (0·29,2·94) | 0·79  0·43  0·31  0·64  0·91  0·68  0·89 |

**Table A7. High Risk of Bias by Overall Risk of Bias (N=300)**

| **Variable** | **Overall Risk of Bias** | |
| --- | --- | --- |
|  | **OR (95%CI)** | ***p-*value** |
| Trial registration | 0·29 (0·12,0·69) | 0·01 |
| Industry funding | 1·28 (0·50,3·29) | 0·61 |
| Multi-centre trial | 2·10 (0·75,5·87) | 0·16 |
| Type of journal  Specialty medical  General medical  Specialty pediatric  General pediatric | Reference  0·49 (0·13,1·91)  3·15 (0·70,14·26)  0·95 (0·30,3·04) | 0·31  0·14  0·93 |
| Nature of intervention  Drug  Device  Natural health product  Vaccine  Other | Reference  1·65 (0·45,6·07)  0·66 (0·20,2·22)  1·81 (0·22,14·81)  5·48 (1·20,24·91) | 0·45  0·50  0·58  0·03 |
| Outcome category  Physiological  Behavioural  Biomarker  Pain  Psychological  Quality of life  Techniques/Training  Other | Reference  3·20 (0·41,25·17)  0·75 (0·23,2·44)  1·74 (0·22,13·97)  1·19 (0·14,9·78)  0·66 (0·03,13·44)  1·98 (0·11,35·61)  0·50 (0·10,2·50) | 0·27  0·64  0·60  0·87  0·79  0·64  0·40 |
